# Color Shift, Color Stability, and Post-Polishing Surface Roughness of Esthetic Resin Composites

**DOI:** 10.3390/ma13061376

**Published:** 2020-03-18

**Authors:** Yara Khalid Alkhadim, Malak Jameel Hulbah, Hani Mohammad Nassar

**Affiliations:** 1 Faculty of Dentistry, King Abdulaziz University, P. O. Box 80209, Jeddah 21589, Saudi Arabia; yalkadem@stu.kau.edu.sa (Y.K.A.); mhulbah0001@stu.kau.edu.sa (M.J.H.); 2 Department of Restorative Dentistry, Faculty of Dentistry, King Abdulaziz University, P. O. Box 80209, Jeddah 21589, Saudi Arabia

**Keywords:** composite resins, color shift, color stability, surface roughness, staining, esthetic resins

## Abstract

The aim of this study was to compare color shift, color stability, and post-polishing surface roughness of esthetic restorative materials. Twenty-five disc-shaped specimens (10 mm in diameter and 2 mm in thickness) from five esthetic resin materials (Z250XT, IPS Empress-Direct, G-ænial, Vit-l-escence, and Ceram.X) were fabricated. Color shift before and immediately after light-curing (∆E_0_) then color stability after immersion in tea, coffee, berry juice, and distilled water were measured using a spectrophotometer. Color changes were measured after 2 (ΔE_2_), 4 (ΔE_4_), 6 (ΔE_6_), and 8 weeks (ΔE_8_). Surface roughness values (Rq) were determined using an optical profilometer after polishing using a rotary polishing system. Data were analyzed using ANOVA and multiple comparison methods at 0.05 significance level. There were no significant differences between the tested materials regarding color shift except between Vit-l-escence and Ceram.X (*p* = 0.033). There was no significant difference between coffee and tea solutions (*p* = 1.0) and between berry juice and distilled water (*p* = 0.15). There was no difference between the tested materials regarding Rq (*p* = 0.057). Ceram.X was associated with the lowest ΔE_8_ values overall. Tested materials were comparable regarding color shift and post-polishing surface roughness. Susceptibility to staining was dependent on the material.

## 1. Introduction

Resin composite restorations are one of the most widely used treatment modality to replace missing tooth structure [1,2,3]. Esthetic applications in the anterior region, such as incisal angle repair and diastema closure, are among the important indications of direct resin restorations. Formulations indicated for the anterior region tend to be nanohybrid resin composites with smaller filler sizes leading to superior optical properties, translucency, and high polishability potential [4,5].

In order for the esthetic restorative materials to be functional, they need to maintain color and shade in order to blend with the neighboring tooth structure. This property is important both in the short term after curing as well as in the long term during function [4,6,7]. Clinically unacceptable color change of resin composites is the primary reason for restoration replacement especially in the anterior region of the oral cavity [8,9]. Among the major reasons for restorations discoloration is the dietary substances consumed by the patient, especially staining beverages such as tea and coffee [10]. These liquids can cause extrinsic staining of the surface of restorations and have been reported previously to affect the perceived shade of the restoration [10,11,12].

Another property that can impact the appearance of esthetic restorations is surface texture; which is directly affected by inorganic fillers of the material [13]. Maintaining smoother surface is required to decrease the accumulation of extrinsic stains on the surface of the restoration and to provide a comfortable feeling when the patient’s tongue comes in contact with the restoration [14,15,16]. In addition, light reflection and surface luster of restorations are directly affected by surface texture.

Although recent formulations of resin composites tend to maintain color and surface smoothness over extended periods of time, they are still prone to staining, discoloration, and changes in surface texture over time. Maintaining these properties is paramount over the lifespan of the restoration. This is a reason that color match and surface texture are two of the criteria assessed by the United States Public Health System (USPHS) to evaluate existing restorations [17]. Major compromise in any of these parameters in anterior esthetic restorations necessitates replacement with the potential of weakening the remaining tooth structure [8]. Hence, choosing an esthetic composite material with adequate color stability and surface texture is vital to avoid frequent replacements and subsequent effects on the remaining tooth structure. Thus, the objective of this project was to evaluate color shift, color stability, and post-polishing surface roughness of esthetic composite materials available in the market after immersion in commonly consumed beverages.

## 2. Materials and Methods

### 2.1. Specimen Preparation

Five types of composite materials: Filtek Z250XT (Z250; 3M ESPE, Dental Products, Saint Paul, MN, USA), IPS Empress Direct (ED; Ivoclar Vivadent, Zurich, Switzerland), G-ænial (GA; GC Dental Products, Tokyo, Japan), Vit-l-escence (VL; Ultradent Products, South Jordan, UT, USA), and Ceram.X (CX; Dentsply; Konstanz, Germany) were used for the study (Table 1). Twenty discs (10 mm in diameter and 2 mm in thickness) from shade A2 of each resin material were fabricated by placing the material in a stainless-steel mold and removing excess material by placing a mylar strip followed by microscopic glass slides on either side of the mold and pressing gently.

### 2.2. Color Shift Determination

Before light curing, each uncured specimen was placed flat on the holding bracket of a spectrophotometer (CE7000A, X-rite, Grand Rapids, MI, USA) in order to record the shade before curing. An area of 8 mm × 3 mm from each specimen was measured three times against a black background after the device was calibrated. The Commission Internationale d’Eclairage (CIE) L*a*b* system data was obtained from the average of three readings of each specimen. After that, each specimen was light cured using a light emitting diode (LED) curing light unit (DemiUltra, Kerr Dental, Orange, CA, USA) for 20 s on either side of the mold. The light curing device was regularly checked for irradiance values to be above 1000 mW/cm^2^ using a digital radiometer (Bluephase Meter II, Ivoclar Vivadent Inc., Amherst, NY, USA). After curing, another shade measurement was done in the spectrophotometer. Color shift after light curing (ΔE_0_) was determined by using the following formula [18]:

(1) $$\Delta E_{0}=\sqrt{{\left(L_{\mathrm{postcure}}-L_{\mathrm{precure}}\right)}^{2}+{\left(a_{\mathrm{postcure}}-a_{\mathrm{precure}}\right)}^{2}+{\left(b_{\mathrm{postcure}}-b_{\mathrm{precure}}\right)}^{2}}=\sqrt{\Delta L^{2}+\Delta a^{2}+\Delta b^{2}}$$

where “precure” and “postcure” parameters were recorded before and after light curing, respectively.

### 2.3. Staining Procedure

Twenty specimens from each composite resin material were placed in each of the following solutions: tea (1 tea bag in 250 mL boiling water simmered for 5 min.; Rabea tea, AMS Baeshen and Co., Jeddah, Saudi Arabia), coffee (15 g of ground coffee in 250 mL of boiling water simmered for 5 min.; Nescafé, Nestlé UK Ltd., Staffordshire, UK), berry juice (200 mL of concentrated mixed berry in 1 L of chilled water; Vimto, Aujan Industries Co., Dammam, Saudi Arabia), and distilled water (n = 5) and stored in an incubator at 37 °C (Memmert, Schwabach, Germany) for 8 weeks with solution replacements every two weeks.

### 2.4. Color Stability after Storage in Beverages

After each 2 weeks of immersion in the staining solutions, specimens were removed from the beverages and gently rinsed with distilled water and dried with an absorbent paper. Specimens’ shades were determined by remeasuring the CIE L*a*b* parameters in the spectrophotometer as described above. Color change after immersion was determined using the following formula:

$$\Delta E_{x}=\sqrt{{\left(L_{\mathrm{staining}\;x\;\mathrm{weeks}}-{\;L}_{\mathrm{postcure}}\right)}^{2}+{\left(a_{\mathrm{staining}\;x\;\mathrm{weeks}}-{\;a}_{\mathrm{postcure}}\right)}^{2}+{\left(b_{\mathrm{staining}\;x\;\mathrm{weeks}}-b_{\mathrm{postcure}}\right)}^{2}}\;=\sqrt{\Delta L^{2}+\Delta a^{2}+\Delta b^{2}}$$

where x indicates the number of weeks of immersion in the staining solutions. ΔE_2_, ΔE_4_, ΔE_6_, and ΔE_8_ were determined representing color changes after 2, 4, 6, and 8 weeks of immersion, respectively. Average ΔE values from specimens were reported for each group at each time point. The result was a 5 × 4 × 4 factorial design with five “material” levels, four “solution” levels, and four “time” points.

### 2.5. Post-Polishing Surface Roughness

Five specimens from each composite material were used to measure surface roughness after polishing. Each specimen was polished using a rotational polishing device (Astropol Polishing System, Ivoclar Vivadent, Zurich, Switzerland) for 15 s for each step in the polishing system using light pressure by one investigator (M.J.H.). Surface roughness values (Rq) were measured using an optical profilometer (Contour GT-K, Bruker, Tucson, AZ, USA) and the average value for each group was reported.

### 2.6. Statistical Testing

Results of color shift and surface roughness data were analyzed by a one-way analysis of variance (ANOVA) test followed by least significant difference (LSD) multiple comparison test. Color stability data for the four time points were analyzed using a mixed model repeated measure ANOVA general linear model (GLM) procedure followed by Bonferroni method to detect statistically significant differences between groups. All statistical analyses were conducted at 5% significance level using SPSS statistical software Ver. 17 (IBM Corporation, Armonk, NY, USA).

## 3. Results

### 3.1. Color Shift (∆E_0_)

**Figure 1:**
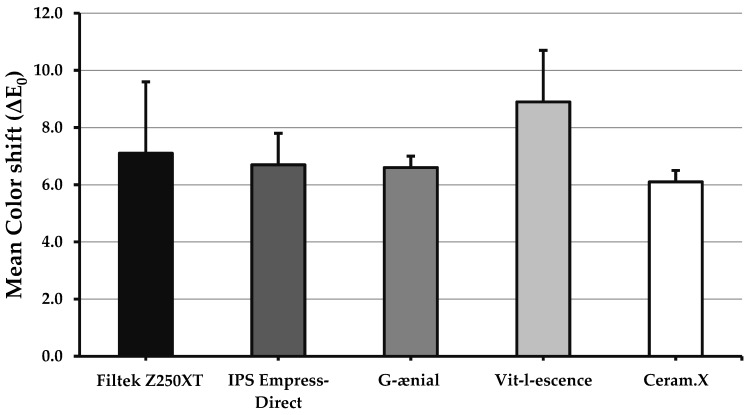
Bar graph showing color shift (∆E_0_) of the investigated materials after light curing. Error bars represent standard error. Only significant difference (*p* < 0.05) was between Vit-l-escense and Ceram.X.

Changes in color before and after light curing (∆E_0_) are presented in Figure 1. The difference of color shift means is statistically significant (*p* = 0.037). The highest and lowest ΔE_0_ values were reported for VL (∆E_0_ = 8.9 ± 3.7) and CX (∆E_0_ = 6.1 ± 2.2), respectively, with a significant difference between the two (*p* = 0.033). Other materials had the same level of color shift after curing, ranging between 6.6 and 7.1.

### 3.2. Color Stability after Storage in Staining Solutions (∆E_2_ − ∆E_8_)

Mean values for color changes after each time point are reported in Table 2. Both tea and coffee produced more significant changes on composite shades compared to berry juice and distilled water (*p* < 0.001); however, no significant difference was reported between tea and coffee (*p* = 1.0) or between berry juice and distilled water (*p* = 0.15).

**Figure 2:**
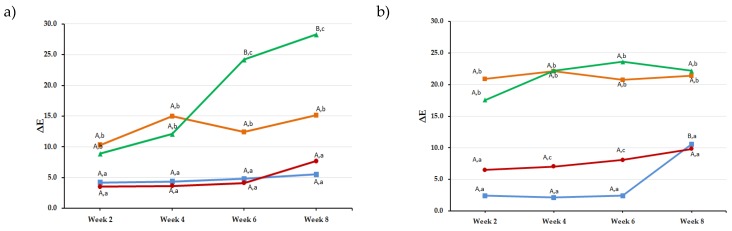

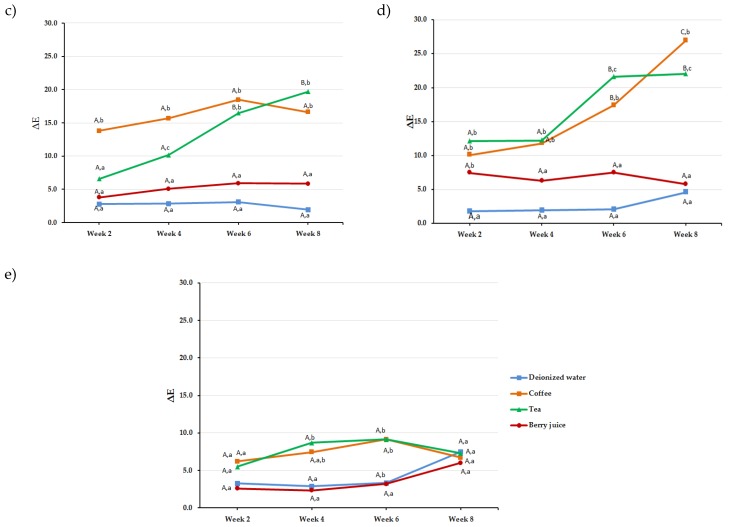
Line graphs showing ΔE values for the five tested materials after immersion in the different solutions for 2, 4, 6, and 8 weeks: (**a**) Filtek Z250XT, (**b**) IPS Empress-Direct, (**c**) G-ænial, (**d**) Vit-l-escence, and (**e**) Ceram.X. Upper case letters indicate statistically significant difference (*p* < 0.05) overtime for the same material for a given solution. Lower case letters indicate statistically significant difference (*p* < 0.05) between solutions at a specific time point for a given material.

The effect of “time” was dependent on the material (Figure 2); however, significant changes were recorded for tea and coffee starting week 2, with subsequently higher ΔE values at subsequent time points.

Regarding “material” effect, all materials where statistically significant from each other except for Z250 compared to GA (*p* = 1.0) and VL (*p* = 0.72), for GA compared to VL (*p* = 0.15), and for ID compared to VL (*p* = 0.4). At 8 weeks, CX had the least ΔE_8_ values among all tested materials with the highest value for tea reported with Z250 (ΔE_8_ = 28.3 ± 4.5) and for coffee with VL (ΔE_8_ = 26.9 ± 8.2).

### 3.3. Post-Polishing Surface Roughness (Rq)

**Figure 3:**
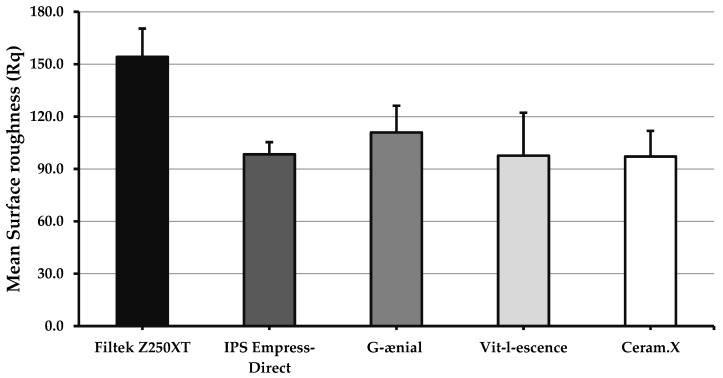
Bar graph showing surface roughness values (Rq) for the five investigated materials after polishing. Error bars represent standard error. No significant difference (*p* ≥ 0.05) between any two materials.

Figure 3 shows values for post-polishing surface roughness (Rq). There was no statistically significant difference between any two types of materials regarding post-polishing surface roughness (*p* = 0.057)

## 4. Discussion

The demand on esthetic restorations is increasing owing to the need for esthetic solutions and preserving tooth structure by avoiding indirect restorations. Although these direct formulations have properties such as translucency, good shade matching, and shade variety in order to blend with tooth structure, multiple factors can affect the color stability of these resin composite formulations, including the type of resin matrix as well as type, size, and amount of fillers [7]. Resin formulations containing nano-sized particles are the materials of choice for direct esthetic applications due to superior optical properties and high polishability potential compared to microhybrid composites [19,20].

Resin composites can be subjected to a variety of sources of staining during their lifespan. Among the frequent sources is the dietary consumption of staining beverages [9,10,11,12]. Further, the inherent properties of the material, such as color shift and the ability to achieve a smooth lustrous surface after polishing, are very important in order to maintain an esthetic result [14,16]. Thus, the objective of the current investigation was to compare five esthetic resin composite formulations available in the market in regard to their color shift, color stability, and post-polishing surface roughness.

In the current investigation, coffee, tea, and berry juice were used since they are common beverages which are frequently consumed throughout the world. We have adopted a continuous immersion approach in order to simulate long-term exposure to staining solutions in the oral cavity. Distilled water was used as a control because previous studies have reported minimal color changes [21]. Still, perceivable color changes were reported in the distilled water groups (Table 2) at the end of the current study possibly affecting the chemistry of the resin material and causing some intrinsic color changes.

To standardize the comparison process between the tested materials, all specimens were fabricated from shade A2 since it is one of the most widely used shades in dental practice owing to its prevalence in human dentition [22]. Shade changes immediately after light curing as well as after immersion in staining solutions were measured using a spectrophotometer in order to yield the CIEL*a*b* parameters. This method is able to detect subtle changes in shades of dental resins and it is a widely used approach to objectively determine color changes in dental restorations expressed as numerical values without the inherent subjectivity of the operator decision-making process [4,11,23,24,25,26,27]. However, all values of color change must be objectively considered since values of ∆E ≥ 3.7 are considered clinically unacceptable [28]. It should be noted that almost all tested materials reported ΔE values larger than 3.7 for coffee and tea after 2 weeks of immersion, and that all ΔE_8_ values of the three tested solutions were above 3.7 (Table 2). This trend is in agreement with previous investigations reporting a time-dependent increase in ΔE values [11,29,30]. Still, the magnitude of color change along with other clinically relevant factors must be considered before the replacement decision is undergone in order to preserve tooth structure.

**Figure 4:**
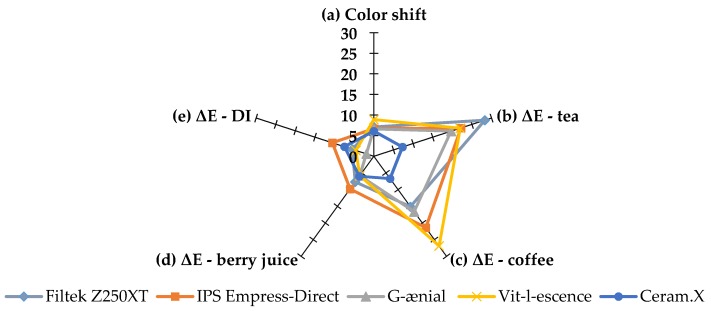
A radar graph showing the performance of the five tested materials on color shift (**a**) directly after curing, color stability after 8 weeks of immersion in (**b**) tea, (**c**) coffee, (**d**) berry juice, and (**e**) deionized water. Point of origin indicates a value of zero and pentagon corners indicate a maximum value of 30 per parameter. Smaller values indicate better esthetic performance.

**Figure 5:**
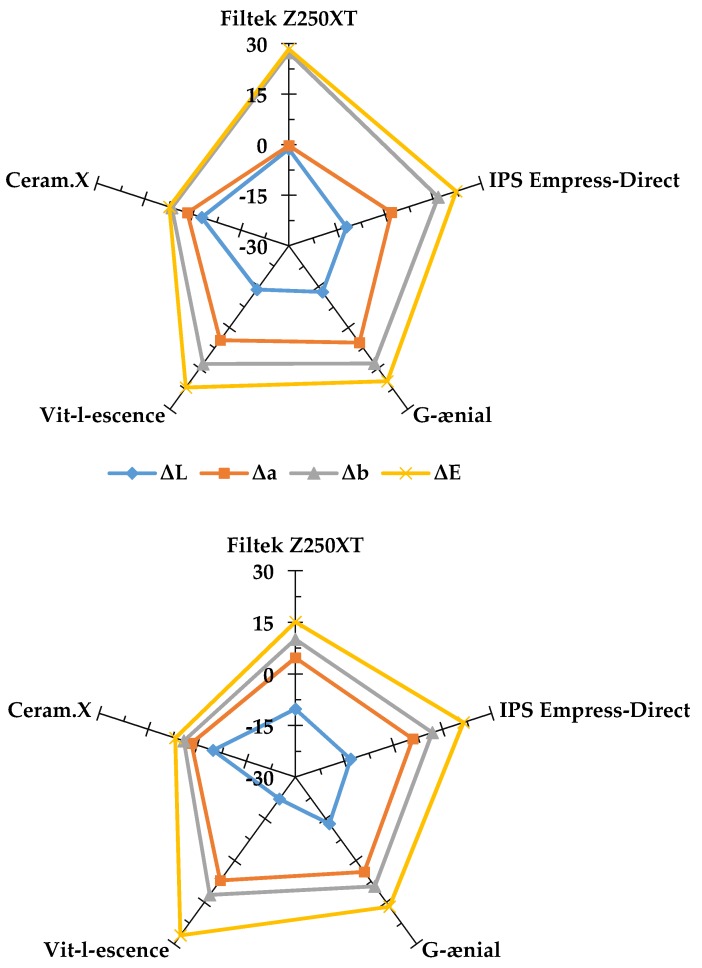
Radar graphs showing the changes in the CIE L*a*b* parameters after 8 weeks of immersion in tea (upper graph) and coffee (lower graph). ΔL: changes in lightness and darkness, Δa: changes in the red–green axis, Δb: changes in the blue–yellow axis, ΔE: overall shade change. Values close to zero indicate better esthetic performance.

The majority of ΔE values can be attributed to positive changes in the b* parameter indicating a yellow color shift in the blue–yellow axis (Table 3). This change was expected in the coffee and tea groups as it has been reported in a previous study [11]; however, the magnitude of the yellow shift was dependent on the material and the duration of immersion reaching a maximum value of Δb = 27.3 ± 3.7 in the Z250 tea group. Further illustration of this effect can be seen in Figure 4 and Figure 5, which show the esthetic performance of the five resin composite materials used in the study in regards to their color shift and stability after 8 weeks in each CIE L*a*b* parameter for tea and coffee. Color shift after light curing was almost the same for all tested materials; however, ΔE_8_ values varied depending on the solution and the material. Z250 was more susceptible to tea, VL was more susceptible to coffee, and CX color changes were the lowest overall compared to other materials. A similar finding was reported by Llena et al. in their study, where a group of materials were stored in red wine, coffee, and cola over a period of 4 weeks [26].

In this investigation, we wanted to compare materials specifically indicated for esthetic purposes. This included two microhybrid (GA and VL), two nanohybrid (Z250 and ED), and one nanoceramic (CX) dental composites. These materials are promoted by their respective manufacturer as esthetic options for anterior restorations. Poggio et al. reported that their tested Filtek formulation was susceptible to color changes especially when immersed in coffee [4]. ΔE values for CX and GA were low compared to other materials in the same investigation. In a previous study, the same authors reported that microhybrid and nanohybrid composites responded similarly when immersed in tea for 14 days [6]. A somewhat similar trend is reported in this investigation (illustrated clearly in Figure 2) with very little difference between microhybrids and nanohybrids expect CX, which is considered a nanoceramic with a novel and different resin matrix chemistry involving polysiloxane, polyurethane methacrylate, and ethoxylated bisphenol-A dimethacrylate (Bis-EMA). It has previously been reported that color stability is affected by the hydrophilicity of resin matrix; hydrophilic resins can attract more water, leading to more stains being picked up which can inversely affect color stability [31,32,33]. Bis-EMA, found in CX, was associated with low hydrophilicity potential leading to less water sorption and, consequently, less uptake of extrinsic stains compared to bisphenolglycidyl methacrylate (Bis-GMA) and urethane dimethacrylate (UDMA) formulations [33,34].

The contribution of each CIE L*a*b* parameter to ΔE_8_ is shown in Figure 5 for both tea and coffee. In both instances, Δb values were highest followed by Δa. A slight darkening effect was reported for all materials immersed in tea and coffee as indicated by negative ΔL values. Although previous studies showed tea as the solution associated with more discoloration in comparison to coffee [11,35,36,37,38], there was no significant difference between tea and coffee in the present investigation as reported previously by Ertas et al. [39]. This difference can be attributed to the immersion methodology but, more importantly, is affected by the nature of the specific materials tested in each investigated as discussed above. Further, a possible difference between coffee and tea is that tea molecules are believed to be able to penetrate deeper into the materials whereas coffee molecules remain on the surface and are more readily removed in distilled water washing step.

Finishing and polishing procedures are essential steps during the placement of direct restorations [40,41]. Similar to factors affecting staining, surface roughness can be affected mainly by filler size; basically, the smaller the size of the filler, the more surface smoothness can be achieved after the polishing process [19]. Although there are multiple finishing and polishing options available, in the current investigation, we wanted to standardize the polishing process in order to compare surface roughness values across the different resin composite brands. We chose Astropol polishing system because it is a versatile kit including all steps required for the finishing and polishing of composite resins. Further, the utilization of an optical-based profilometer provided more reliable surface topography measurements [42].

Newly esthetic composite restoration involves small particle filler sizes ranging from 0.01 to 0.04 µm, which is helpful in improving the physical properties of esthetic restoration such as by providing better optical characteristics, better glossy shiny surface, and reduced polymerization shrinkage [43]. The main indications for finishing and polishing are removing excess material and surface roughness of restoration, which are helpful for improving patient comfort, maintain healthy soft tissues, and ensuring good surface resistance to discoloration by decreasing the risk for uptake of stains [7,14,15,40,44]. Hybrid composites such as Herculite XRV have large particle sizes compared to nanocomposites, leading to high surface roughness after finishing and polishing with an increased risk of surface discoloration [45]. However, in the present investigation, there were no differences in post-polishing surface roughness (Rq = 97.1 − 110.1) despite differences in filler sizes (*p* = 0.057). This could possibly translate into comparable performance in the oral cavity with equivalent stain uptake and plaque accumulation potentials.

As with other in vitro investigations, this study has some limitations. The laboratory setting does not fully simulate oral conditions such as the presence of saliva, pH challenges, and abrasion by mastication and oral hygiene practices [46]. Further, the geometrical shape of the fabricated specimens does not resemble typical dental restorations. Still, current findings can be useful in extrapolating in vivo behavior of the tested materials and guide future clinical studies.

## 5. Conclusions

In conclusion, the different esthetic materials tested did not have major differences regarding color shift and surface roughness after polishing using a rotary system. The staining potential of coffee and tea were comparable, and the effect increased with time. Further, the intensity of the staining was dependent on the material. Overall, Ceram.X was associated with high color stability, whereas other materials showed more color changes, especially IPS Empress-Direct.

## Figures and Tables

**Table 1:** Summary of the products used in the study.

Material, Abbreviation	Category	Resin Matrix	Main Fillers Type and Size	Filler Load (wt.%/vol.%)	Manufacturer
Filtek Z250XT (Z250)	Nanohybrid	Bis-GMA, UDMA, Bis-EMA, PEGDMA, TEGDMA	Zirconia and silica (0.02–0.6 µm)	78/68	3M ESPE, Dental Products, Saint Paul, Minnesota, USA
IPS Empress-Direct (ED)	Nanohybrid	UDMA, Bis-GMA, TEGDMA	Barium glass, ytterbium trifluoride, and mixed oxides (0.5 µm)	75/52	Ivoclar Vivadent, Zurich, Switzerland
G-ænial (GA)	Microhybrid	UDMA, dimethacrylate co-monomers	Silica, strontium, lanthanoid fluoride, fumed silica (0.1–17 µm)	76/50	GC Dental Products, Tokyo, Japan
Vit-l-escence (VL)	Microhybrid	Bis-GMA, TEGDMA	Silica (0.7 µm)	75/52	Ultradent Products, South Jordan, Utah, USA
Ceram.X (CX)	Nanoceramic	Methacrylate-modified polysiloxane, polyurethane methacrylate, Bis-EMA, TEGDMA	Barium-aluminum borosilicate glass, methacrylate functionalized silicon dioxide (0.01–1.5 µm)	77/59	Dentsply, Konstanz, Germany

**Table 2:** Mean values of color change (ΔE_2_ − ΔE_8_) of the five restorative materials after exposure to the four beverages (n = 5). Color change after 2 (ΔE_2_), 4 (ΔE_4_), 6 (ΔE_6_), and 8 weeks (ΔE_8_).

Material	Solution	ΔE_2_	ΔE_4_	ΔE_6_	ΔE_8_
Filtek Z250XT	Tea	8.9 ± 4.1 ^A,b^	12.1 ± 4.4 ^A,b^	24.0 ± 1.9 ^B,c^	28.3 ± 4.5 ^B,c^
	Coffee	10.3 ± 2.2 ^A,b^	15.0 ± 5.7 ^A,b^	12.4 ± 4.5 ^A,b^	15.1 ± 3.0 ^A,b^
	Berry juice	3.5 ± 1.2 ^A,a^	3.7 ± 1.0 ^A,a^	4.1 ± 2.3 ^A,a^	7.7 ± 6.2 ^A,a^
	Distilled water	4.2 ± 6.5 ^A,a^	4.4 ± 6.3 ^A,a^	4.8 ± 4.7 ^A,a^	5.5 ± 7.6 ^A,a^
IPS Empress Direct	Tea	17.6 ± 8.0 ^A,b^	22.2 ± 3.4 ^A,b^	23.6 ± 5.9 ^A,b^	22.2 ± 6.0 ^A,b^
	Coffee	20.9 ± 4.9 ^A,b^	22.1 ± 2.4 ^A,b^	20.8 ± 4.8 ^A,b^	21.4 ± 2.9 ^A,b^
	Berry juice	6.5 ± 3.0 ^A,a^	7.0 ± 3.1 ^A,c^	8.1 ± 1.6 ^A,c^	9.8 ± 1.7 ^A,a^
	Distilled water	2.5 ± 0.6 ^A,a^	2.2 ± 1.2 ^A,a^	2.4 ± 0.5 ^A,a^	10.6 ± 5.5 ^B,a^
G-ænial	Tea	6.6 ± 0.5 ^A,a^	10.2 ± 4.0 ^A,c^	16.5 ± 2.3 ^B,b^	19.7 ± 4.7 ^B,b^
	Coffee	13.8 ± 0.6 ^A,b^	15.7 ± 1.9 ^A,b^	18.5 ± 1.1 ^A,b^	16.6 ± 0.9 ^A,b^
	Berry juice	3.8 ± 0.8 ^A,a^	5.1 ± 0.8 ^A,a^	5.9 ± 2.5 ^A,a^	5.8 ± 1.2 ^A,a^
	Distilled water	2.8 ± 0.5 ^A,a^	2.9 ± 1.0 ^A,a^	3.1 ± 0.9 ^A,a^	1.9 ± 0.7 ^A,a^
Vit-l-escence	Tea	12.1 ± 6.8 ^A,b^	12.2 ± 2.4 ^A,b^	21.6 ± 7.2 ^B,c^	22.0 ± 6.9 ^B,c^
	Coffee	10.1 ± 1.3 ^A,b^	11.8 ± 2.9 ^A,b^	17.4 ± 4.0 ^B,b^	26.9 ± 8.2 ^C,b^
	Berry juice	7.5 ± 4.7 ^A,b^	6.3 ± 4.3 ^A,a^	7.5 ± 4.9 ^A,c^	5.8 ± 3.7 ^A,a^
	Distilled water	1.8 ± 1.1 ^A,a^	2.0 ± 1.3 ^A,a^	2.1 ± 0.8 ^A,a^	4.6 ± 1.8 ^A,a^
Ceram.X	Tea	5.5 ± 2.6 ^A,a^	8.7 ± 4.2 ^A,b^	9.2 ± 2.2 ^A,b^	7.3 ± 2.0 ^A,a^
	Coffee	6.2 ± 3.2 ^A,a^	7.5 ± 4.2 ^A,a,b^	9.2 ± 1.8 ^A,b^	6.7 ± 4.3 ^A,a^
	Berry juice	2.6 ± 0.7 ^A,a^	2.4 ± 1.5 ^A,a^	3.2 ± 1.7 ^A,a^	6.0 ± 2.0 ^A,a^
	Distilled water	3.3 ± 1.5 ^A,a^	2.9 ± 1.1 ^A,a^	3.4 ± 1.0 ^A,a^	7.5 ± 1.3 ^A,a^

Upper case letters indicate statistically significant difference (*p* < 0.05) overtime for the same material for a given solution. Lower case letters indicate statistically significant difference (*p* < 0.05) between solutions at a specific time point for a given material.

**Table 3:** Means and standard deviations of CIE Lab parameters for the materials tested in the four solutions after 8 weeks of immersion. ΔL: changes in lightness and darkness, Δa: changes in the red–green axis, Δb: changes in the blue–yellow axis, ΔE: overall shade change.

Material	Solution	ΔL	Δa	Δb	ΔE
Filtek Z250XT	Tea	−1.3 ± 8.5	−0.3 ± 2.1	27.3 ± 3.7	28.3 ± 4.5 *
	Coffee	−10.2 ± 2.6	4.7 ± 1.8	10.1 ± 1.3	15.1 ± 3.0 *
	Berry juice	−3.4 ± 8.3	1.8 ± 1.0	4.2 ± 1.0	7.7 ± 6.2 *
	Distilled water	4.7 ± 8.1	0.4 ± 0.3	−1.0 ± 1.3	5.5 ± 7.6 *
IPS Empress-Direct	Tea	−12.0 ± 9.1	2.0 ± 4.0	16.6 ± 4.8	22.2 ± 6.0 *
	Coffee	−13.1 ± 3.9	5.9 ± 1.1	11.9 ± 2.5	21.4 ± 2.9 *
	Berry juice	0.5 ± 6.0	3.3 ± 0.5	7.6 ± 1.1	9.8 ± 1.7 *
	Distilled water	10.5 ± 5.4	0.6 ± 0.2	−0.8 ± 0.9	10.6 ± 5.5 *
G-ænial	Tea	−13.0 ± 5.4	5.6 ± 2.6	13.2 ± 1.5	19.7 ± 4.7 *
	Coffee	−13.2 ± 1.0	4.1 ± 0.4	9.3 ± 0.5	16.6 ± 0.9 *
	Berry juice	−2.9 ± 1.2	2.0 ± 0.7	4.5 ± 1.3	5.8 ± 1.2 *
	Distilled water	0.1 ± 1.4	0.2 ± 0.1	−1.4 ± 0.9	1.9 ± 0.7
Vit-l-escence	Tea	−13.9 ± 9.8	4.7 ± 5.7	13.4 ± 5.8	22.0 ± 6.9 *
	Coffee	−22.1 ± 9.8	7.2 ± 2.8	12.4 ± 1.6	26.9 ± 8.2 *
	Berry juice	12.8 ± 20.6	0.6 ± 1.3	3.0 ± 3.5	5.8 ± 3.7 *
	Distilled water	4.5 ± 1.8	0.1 ± 0.2	0.6 ± 0.6	4.6 ± 1.8 *
Ceram.X	Tea	−2.8 ± 1.5	1.7 ± 0.5	6.5 ± 1.7	7.3 ± 2.0 *
	Coffee	−4.8 ± 3.9	1.8 ± 0.9	4.1 ± 2.1	6.7 ± 4.3 *
	Berry juice	4.0 ± 2.8	1.2 ± 0.4	3.7 ± 1.7	6.0 ± 2.0 *
	Distilled water	7.4 ± 1.2	0.6 ± 0.2	0.3 ± 1.2	7.5 ± 1.3 *

* Change of ΔE ≥ 3.7 is considered visually detectable.

## Abbreviations

Bis-GMA	bisphenolglycidyl methacrylate
Bis-EMA	ethoxylated bisphenol-A dimethacrylate
PEGDMA	polyethylene glycol dimethacrylate
TEGDMA	triethylene glycol dimethacrylate
UDMA	urethane dimethacrylate
wt.%	weight percentage
vol.%	volume percentage
